# Do immunosuppressive treatments influence immune responses against adenovirus-based COVID-19 vaccines in patients with multiple sclerosis? An Argentine multicenter study

**DOI:** 10.3389/fimmu.2024.1431403

**Published:** 2024-08-19

**Authors:** Berenice Anabel Silva, Esteban Miglietta, Juan Cruz Casabona, Shirley Wenker, María Bárbara Eizaguirre, Ricardo Alonso, Magdalena Casas, Luciana Grimanesa Lázaro, Federico Man, Gustavo Portuondo, Abril Lopez Bisso, Noelia Zavala, Federico Casales, Gastón Imhoff, Dra Judith Steinberg, Pablo Adrián López, Edgar Carnero Contentti, Norma Deri, Vladimiro Sinay, Javier Hryb, Edson Chiganer, Felisa Leguizamon, Verónica Tkachuk, Johana Bauer, Flavia Ferrandina, Susana Giachello, Paula Henestroza, Orlando Garcea, Carla Antonela Pascuale, Mauro Heitrich, Osvaldo L. Podhajcer, Sabrina Vinzón, Tomas D’Alotto-Moreno, Alejandro Benatar, Gabriel Adrián Rabinovich, Fernando J. Pitossi, Carina C. Ferrari

**Affiliations:** ^1^ Multiple Sclerosis Unit, Italian Hospital of Buenos Aires, Buenos Aires, Argentina; ^2^ Laboratorio de Terapias Regenerativas y Protectoras del Sistema Nervioso, Fundación Instituto Leloir, Instituto de Investigaciones Bioquímicas de Buenos Aires (IIBBA), Consejo Nacional de Investigaciones Científicas y Técnicas (CONICET), Buenos Aires, Argentina; ^3^ Centro Universitario de Esclerosis Múltiple, Hospital Ramos Mejía, Buenos Aires, Argentina; ^4^ Carrera del Personal de Apoyo (CPA), Instituto de Investigaciones Bioquímicas de Buenos Aires (IIBBA), Consejo Nacional de Investigaciones Científicas y Técnicas (CONICET), Fundación Instituto Leloir, Buenos Aires, Argentina; ^5^ Neurology Deparment, Sanatorio de los Arcos, Buenos Aires, Argentina; ^6^ Neurology Deparment, Hospital Británico de Buenos Aires, Buenos Aires, Argentina; ^7^ Neurosciences Deparment, Hospital Alemán, Buenos Aires, Argentina; ^8^ Multiple Sclerosis Unit, Instituto de Asistencia Integral en Diabetes y patologías crónicas (DIABAID), Buenos Aires, Argentina; ^9^ Multiple Sclerosis Deparment, Fundación Favaloro, Hospital Universitario, Buenos Aires, Argentina; ^10^ Neurology Deparment, Hospital General de Agudos Carlos G. Durand, Buenos Aires, Argentina; ^11^ Neurology Deparment, Hospital General de Agudos Dr. Teodoro Álvarez, Buenos Aires, Argentina; ^12^ Neurology Deparment, Hospital de Clínicas José de San Martín, Buenos Aires, Argentina; ^13^ Asociación Esclerosis Múltiple Argentina, Buenos Aires, Argentina; ^14^ Asociación Lucha Contra la Esclerosis Múltiple, Buenos Aires, Argentina; ^15^ Laboratorio de Terapias Moleculares y Celulares, Instituto de Investigaciones Bioquímicas de Buenos Aires (IIBBA), Consejo Nacional de Investigaciones Científicas y Técnicas (CONICET), Fundación Instituto Leloir, Buenos Aires, Argentina; ^16^ Laboratorio de Glicomedicina, Instituto de Biología y Medicina Experimental (IBYME), Buenos Aires, Argentina

**Keywords:** multiple sclerosis, vaccine, immune response, COVID - 19, cell response, Omicron (BA.1)

## Abstract

**Introduction:**

There are no reports in LATAM related to longitudinal humoral and cellular response to adenovirus based COVID-19 vaccines in people with Multiple Sclerosis (pwMS) under different disease modifying therapies (DMTs) and neutralization of the Omicron and Wuhan variants of SARS-COV-2.

**Methods:**

IgG anti- SARS-COV-2 spike titer were measured in a cohort of 101 pwMS under fingolimod, dimethyl fumarate, cladribine and antiCD20, as well as 28 healthy controls (HC) were measured 6 weeks after vaccination with 2^nd^ dose (Sputnik V or AZD1222) and 3^nd^ dose (homologous or heterologous schedule). Neutralizing capacity was against Omicron (BA.1) and Wuhan (D614G) variants and pseudotyped particles and Cellular response were analyzed.

**Results:**

Multivariate regression analysis showed anti-cd20 (β= -,349, 95% CI: -3655.6 - -369.01, p=0.017) and fingolimod (β=-,399, 95% CI: -3363.8 - -250.9, p=0.023) treatments as an independent factor associated with low antibody response (r^2^ adjusted=0.157). After the 2nd dose we found a correlation between total and neutralizing titers against D614G (rho=0.6; p<0.001; slope 0.8, 95%CI:0.4-1.3), with no differences between DMTs. Neutralization capacity was lower for BA.1 (slope 0.3, 95%CI:0.1-0.4). After the 3rd dose, neutralization of BA.1 improved (slope: 0.9 95%CI:0.6-1.2), without differences between DMTs. A fraction of pwMS generated anti-Spike CD4+ and CD8+ T cell response. In contrast, pwMS under antiCD20 generated CD8+TNF+IL2+ response without differences with HC, even in the absence of humoral response. The 3rd dose significantly increased the neutralization against the Omicron, as observed in the immunocompetent population.

**Discussion:**

Findings regarding humoral and cellular response are consistent with previous reports.

## 1 Introduction

Severe acute respiratory syndrome coronavirus 2 (SARS-CoV-2), the virus that causes coronavirus disease 2019 (COVID-19), has led to a serious global health crisis, resulting in high rates of illness and death (1). Vaccination is seen as the most effective and safest way to protect people against this disease with several vaccines available in Argentina. People with multiple sclerosis (pwMS) are considered high risk and should be particularly prioritized for vaccination (2). However, certain disease-modifying therapies (DMTs) for MS like immunomodulators and immunosuppressants can cause T- and/or B-cell depletion and may reduce immune responses to COVID-19 (3, 4), especially in the case of anti-CD20 therapies (ocrelizumab and rituximab) and fingolimod, which can cause lymphopenia (5–9).

To date, there is a significant amount of data from Europe and the USA regarding the immune response to COVID-19 vaccines in pwMS who are undergoing specific treatments. Most studies focus on RNA-based or inactivated virus vaccines, revealing that approximately 50% of patients on fingolimod and approximately 80% of patients on anti-CD20 treatment do not produce significant immune responses in their initial vaccination (10–20).

This has raised questions for neurologists worldwide including whether pwMS with lower antibody production should receive additional booster shots and if treatment should be adjusted based on the vaccine type. Limited information exists on the immune response in Latin American patients with MS who predominantly received adenovirus-based vaccines. Notably, Sputnik V has been applied in few countries in the world, including Argentina.

Therefore, our study aims to investigate the immune response to these vaccines in pwMS undergoing anti-CD20 (ocrelizumab and rituximab), fingolimod, cladribine, and dimethyl fumarate treatments compared to the general population. Additionally, we will assess the safety of these vaccines.

## 2 Methods

### 2.1 Human subjects and study design

A multicenter prospective/longitudinal study was conducted from the Multiple Sclerosis University Center (CUEM) of the JM Ramos Mejía Hospital in Buenos Aires. Peripheral blood was collected from all the participants at the hospital 6–8 weeks after the second and third doses of the COVID-19 vaccine. The study involved healthy controls (HC) and pwMS according to the McDonald 2017 criteria (21) from nine specialized MS centers. Inclusion criteria were adults over 18 years old who received the first two doses of the Sputnik V and/or Oxford-AstraZeneca (AZD1222) vaccines against COVID-19, and/or combinations of both, with an interval of more than 1 month between doses. It should be noted that, in our country, owing to the lack of vaccines, vaccination schemes were carried out with time intervals between doses greater than the 21-day minimum indicated in the vaccine insert.

The exclusion criteria included HC/pwMS who did not wish to participate in the study, HC/pwMS under 18 years old, HC/pwMS vaccinated with the first two doses of the Sputnik V and Oxford-AstraZeneca vaccines with a 21-day interval, recipients of Sinopharm vaccine, and those with a history of COVID-19 disease. No baseline antibody titers were measured before vaccination.

The data collected from both pwMS/HC included age, sex, type of vaccine, and date of vaccination. For pwMS, additional information included age at MS symptom onset, age at MS diagnosis, years of disease evolution, DMT used, lymphocyte count at vaccination, current Expanded Disability Status Scale (EDSS), and vaccine-related adverse events. Symptoms post-vaccination, as per Achiron et al. (2021), were recorded following a predefined list including pain at injection site, fever, muscle/joint pain, flu-like symptoms, fatigue/weakness, headache, dizziness, gastrointestinal issues (nausea, vomiting, and diarrhea), face tingling, facial weakness, acute MS relapse, and worsening of MS symptoms. For patients on anti-CD20 treatment, the date of last infusion before vaccination was noted, and for those receiving cladribine, the date of the last treatment cycle before vaccination was recorded.

### 2.2 Humoral and cellular response

Serum samples were obtained 6–8 weeks after receiving the vaccine in all participants. They were processed at the Fundación Instituto Leloir, where the titers of IgG antibodies against the spike protein were evaluated by ELISA, the COVIDAR IgG kit, created in our country by colleagues (“COVIDAR” Platform) (22).

A Pseudo Virus Based Neutralization Assay was performed in order to assess the ability of the sera to neutralize different SARS-CoV-2 variants of concern. Basically, pseudo viral particles (PVs) containing SARS-CoV-2 Spike-D614G or SARS-CoV-2 Spike-BA.1 (Omicron) protein were generated as previously described (23). We generated a replication defective vesicular stomatitis virus (VSV) PV in which the backbone was provided by a pseudo-typed ΔG-luciferase (G*ΔG-luciferase) rVSV (Kerafast, Boston, MA, USA) that packages the expression cassette for firefly luciferase instead of VSV-G in the VSV genome. The 50% tissue culture infectious dose (TCID_50_) of SARS-CoV-2 PV was determined in sextuplicates and calculated using the Reed–Muench method as previously described (24).

The neutralization assays were performed as previously described (23). Briefly, 50 µl of serially diluted sera was combined with 65 TCID_50_ PVs in 50 µl of complete medium (DMEM supplemented with 10% FBS and non-essential amino acids) in 96-well plates (Greiner Bio-One, Germany) and incubated at 37°C, 5% CO_2_ for 1 h. Next, 100 µl of 5 × 10^5^/mL HEK293T-ACE2 cells were added to the pseudo virus–serum mixture and incubated at 37°C, 5% CO_2_ for 20–24 h. Conditions were tested in duplicate wells on each plate, and a virus control (VC = no sera) and cell control (CC = no PV) were included on each plate in six wells each to determine the value for 0% and 100% neutralization, respectively. Media was then aspirated from cells and Firefly luciferase activity was determined with the Luciferase Assay System (Promega) as recommended by the manufacturer. The percentage of inhibition of infection for each dilution of the sample is calculated according to the RLU values as follows: % inhibition = [1 – (average RLU of sample – average RLU of CC)/(average RLU of VC – average RLU of CC)] × 100%. On the basis of these results, the ID_50_ of each sample was calculated by the Reed–Muench method.

In order to analyze cellular immune response by flow cytometry, cryopreserved PBMCs (−80°C) were thawed, washed in RPMI 1640 (Serendipia, Buenos Aires, Argentina) supplemented with 10% FBS (Sigma, St. Louis, MO, USA) (complete RPMI), resuspended in PBS, and incubated for 10 min in the presence of 0.1 mg/mL of DNase I (Roche, Basel, Switzerland). Cells were plated in each well of a P96-well plate, incubated in 100 µl of complete RPMI overnight at 37°C, 5% CO_2_, and then cultured in the presence of 1 µg/mL SARS-CoV-2-specific peptides pools (Miltenyi, Bergisch Gladbach, Germany) for 6 h. Negative controls (cultures in the absence of peptides) were included as well as a positive control stimulated with phorbol-12-myristate-13-acetate (PMA) and ionomycin. In all treatments, Brefeldin A and Monensin (BioLegend, San Diego, CA, USA) were added to cultures for the last 4 h.

Cells were washed, incubated with Zombie Violet viability probe (BioLegend, San Diego, CA, USA), surface stained (CD3-BV510 Clone: OKT3, CD4-APCCy7 Clone: OKT4, CD8-PE Clone: RPA-T8, CD45RO-BV785 Clone: UCHL1), fixed with 1% paraformaldehyde (Sigma, St. Louis, MO, USA), then permeabilized and intracellularly stained (CD154-FITC Clone: 24-31, IFN-γ-PerCP-Cy5.5 Clone: 4S.B3, TNF-α-PECy7 Clone: MAb11, and IL-2-APC Clone: MQ1-17H12) in perm wash solution (BioLegend, San Diego, CA, USA). Each incubation step was performed at room temperature for 25 min in darkness. Fluorescent monoclonal antibodies were all purchased from BioLegend (San Diego, CA, USA). Samples were acquired on a BD LSR Fortessa™ X-20 Flow Cytometer and analyzed with FlowJo software (BD, USA). CD4+ and CD8+ lymphocyte cells were analyzed separately after excluding doublets and death cells. TNF and IL-2 parameters and TNF, CD154, IL-2, and IFN-γ were analyzed for CD8+ and CD4+ cells, respectively. Percentage of double positives and simple positives were considered to assess T-cell response. Samples with a very low number of CD4+ and CD8+ cells were excluded from analysis as well as samples that were non-reactive to PMA/ionomycin.

**Figure 1:**
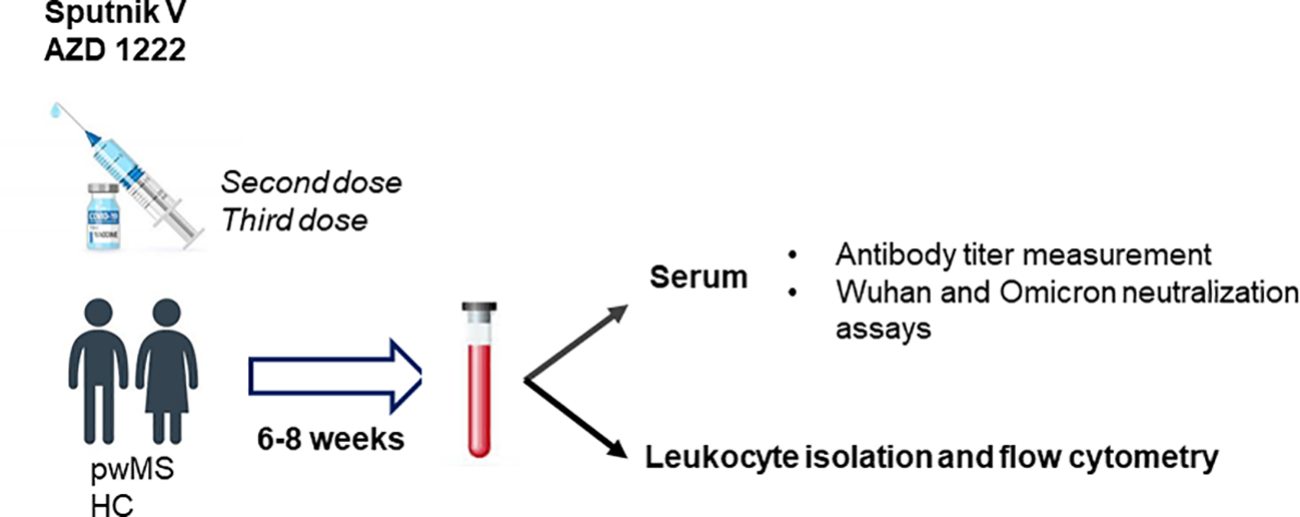
Methods. IgG anti-SARS-COV-2 spike titers and Wuhan and Omicron neutralization assays were performed in serum samples of a cohort of people with Multiple Sclerosis (pwMS) under immunosuppressive treatment and healthy controls (HC) between 68 weeks after second and third dose of Sputnik V and AZD 122 vaccines. Furthermore, leukocyte isolation and flow cytometry were carry out in order to assessed cellular immune response post-vaccine.

The methods are summarized in **Figure 1**.

### 2.3 Ethical aspects

This protocol was approved by the Ethics Committee of the participating centers. All HC and pwMS who were involved were invited to take part in the study and signed the corresponding informed consent if they chose to participate. All samples were properly anonymized.

### 2.4 Statistical analysis

Data were analyzed using the GraphPad Prism 8 statistical package. A descriptive analysis of all the variables included was conducted. Pearson and Spearman correlation tests were employed to analyze the correlation between numerical variables, based on the normality of the distribution of the population. The Student’s *t*-test was utilized for assessing heterogeneity between independent groups as a parametric test, while the Mann–Whitney *U*-test served as the non-parametric test, or ANOVA test was used depending on the number of categorical variables. Significance was defined as a probability of less than or equal to 5% (*p* < 0.05). The effect of the treatments on antibody response was evaluated using the Kruskal–Wallis test and its *post-hoc* test, incorporating HC in this analysis. Additionally, a multivariate analysis was carried out with the antibody titer as the dependent variable and demographic/clinical variables as independent variables. The comparison of different treatments and dose effect of Wuhan/BA.1 variant neutralization was adjusted using linear regression with the correlation calculated through Spearman’s non-parametric test.

## 3 Results

### 3.1 Antibody-mediated immune responses to the first two vaccine doses (primary regimen) in patients with pwMS and HC

A total of 101 pwMS and 28 HC were included. Among them, 35.7% (*n* = 46) were vaccinated with Sputnik V, 51.9% (*n* = 67) were vaccinated with AZD1222, and 12.4% (*n* = 16) were vaccinated with a combination of both vaccines. The interval between doses was 60.1 ± 6.3 days for the AZD1222 vaccine, 102.2 ± 8.5 days for Sputnik V, and 99.8 ± 10.4 for the combined schedule. None of the participants, whether pwMS or HC, had COVID-19 before vaccination. The baseline characteristics of the included population are summarized in **Table 1**.

**Table 1:** Demographic and clinical characteristics of people with Multiple Sclerosis and healthy controls included in the study.

Variable	PwMS (*n* = 101)	HC (*n* = 28)	*p*
Sex (%, *n*)			*p* = 0.3
Female Male	61.4% (62) 28.6% (39)	57.1% (16) 42.9% (12)	
Age (mean, SD)	36.9 (± 11.4)	35% (± 7.9)	*p* = 0.7
MS phenotype (%, *n*)	RR 93.06% (94) PP 6.93% (7)	NA	
EDSS (mean, SD)	2.4 (± 1.5)	NA	
MS evolution, years (mean, SD)	7.6 (± 5.1)	NA	
Vaccine type (%, *n*)			*p* = 0.42
Sputnik V AZD1222 Combined schedule	37.6% (38) 50.5% (51) 11.9% (12)	28.6% (8) 57.1%(16) 14.3% (4)	
Interval between doses (mean, SD)	81 (± 24.1)	81.07 (± 22.2)	*p* = 0.8
DMT (%, *n*)	Dimethylfumarate 22.7% (23) Fingolimod 44.5%(45) Cladribine 13.8% (14) Anti-CD20* 18.8% (19)	NA	

PwMS, people with multiple sclerosis; HC, healthy controls; SD, standard deviation; EDSS, Expanded Disability Status Scale; DMT, disease-modifying therapy; *includes ocrelizumab and rituximab.

Both pwMS and HC were similar in terms of sex, age, vaccine type, and interval between doses. In terms of anti-Spike antibody response, all HC produced antibodies compared to 32.7% (*n* = 33) of pwMS who did not seroconvert. In the pwMS group, there was no association between absence of seroconversion and the type of vaccine received (chi-square = 0.3, *p* = 0.8). The influence of DMTs was also examined, revealing that 100% of pwMS treated with dimethylfumarate and cladribine were able to produce a detectable humoral immune response, while 42.2% (*n* = 19) of pwMS under fingolimod treatment and 73.6% (*n* = 14) with anti-CD20 did not elicit detectable anti-Spike antibodies. A statistically significant association between treatment type and antibody response was observed (chi-square = 34.3, *p* = 0.04) (**Table 2**).

**Table 2:** Humoral immune response to adenovirus-based COVID-19 vaccines according to disease-modifying treatment received.

DMT	PwMS without antibody response, *n* (%)	PwMS with antibody response, *n* (%)	Total number
Fingolimod	19 (42.2)	26 (57.8)	45 (100)
Cladribine	0 (0)	14 (100)	14 (100)
Anti-CD20*	14 (73.6)	5 (26.4)	19 (100)
Dimethylfumarate	0 (0)	23 (100)	23 (100)

We found a statistically significant association between the treatment received and the generation of the antibody response (chi square = 34.3, p = 0.04) (contingency table). PwMS, people with multiple sclerosis; DMT, disease-modifying therapy, *includes ocrelizumab and rituximab.

**Figure 2:**
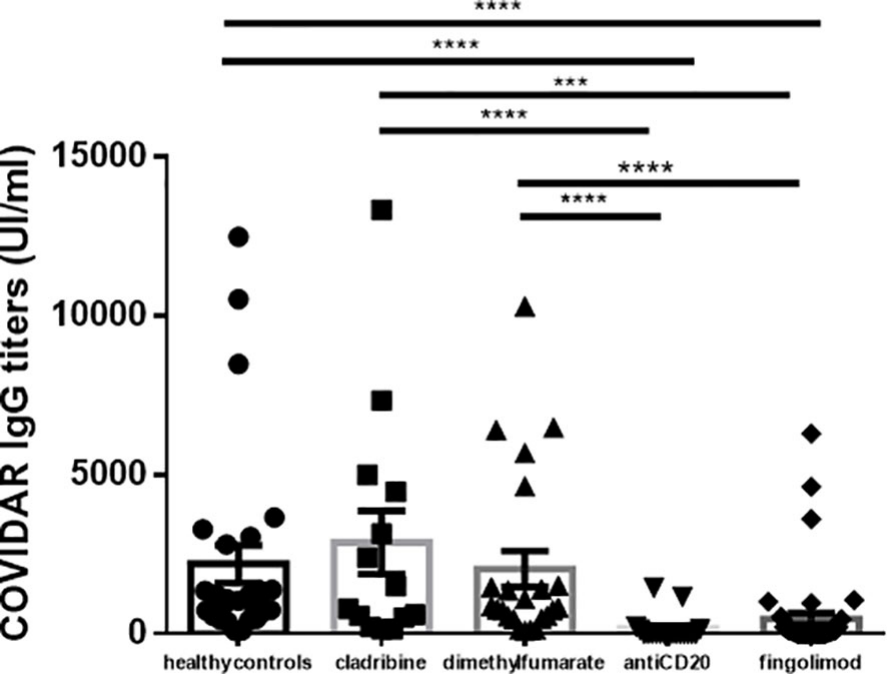
Antibody titers obtained by disease modifying therapy. We found a significantly decreased in the antibody titers in the pwMS under fingolimod and antiCD20 treatments, compared to the control group and the other treatments (p <0.0001, one-way ANOVA). ***p < 0.0001-0.001, ****p < 0.0001. HC, healthy controls; SD, standard deviation; pwMS, people with Multiple Sclerosis.

Furthermore, a significant decrease in anti-Spike IgG antibody titers was observed in pwMS undergoing treatment with fingolimod and anti-CD20, compared to HC or other patients with MS undergoing DMT (*p* < 0.0001, one-way ANOVA) (**Figure 2**).

**Figure 3:**
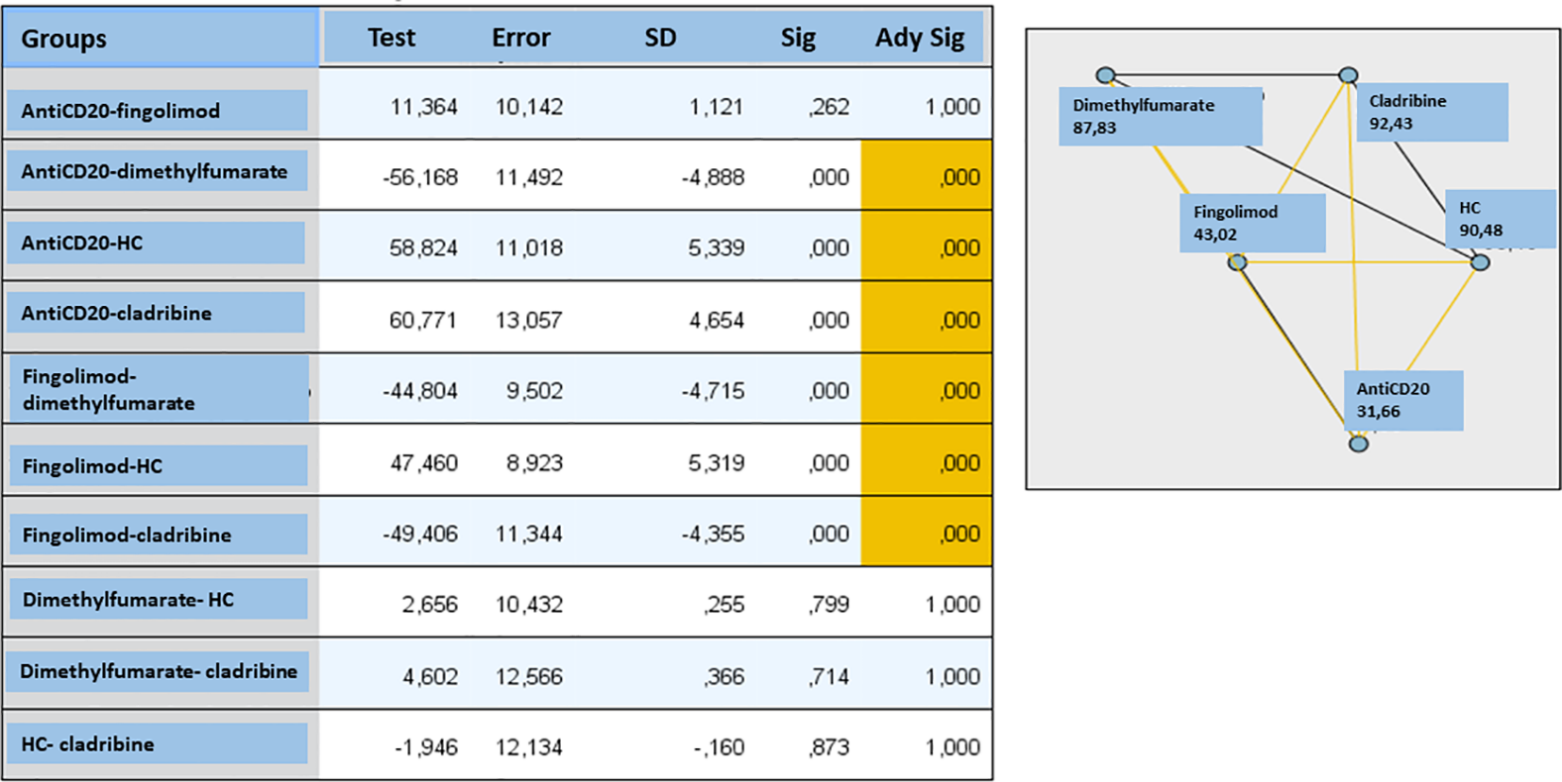
Effect of multiple sclerosis treatments on antibody response analyzing by Kruskal-Wallis test. We found statistically significant differences in antibody titers between the different treatment groups (H(4) = 60.8, p < 0.01) In the post hoc comparisons, statistically significant differences were observed between the treatment pairs analyzed in which one of them was fingolimod or antiCD20 ( p < 0.0001 in all pairs, indicated in the table and in the graph in yellow), SD, standard deviation; HC, healthy controls; sig, significance.

Additionally, we analyzed the effect of the DMT on the antibody response. Statistically significant differences in antibody titers were found between groups [*H* (4) = 60.8, *p* < 0.01] (Kruskal–Wallis). *Post-hoc* analyses showed significant differences between treatment pairs involving fingolimod or anti-CD20: *p* < 0.0001 for all of them (**Figure 3**).

The impact of fingolimod or anti-CD20 treatment on the antibody response did not vary based on the vaccination schedule used (chi-square = 0.65, *p* = 0.7; chi-square = 0.8, *p* = 0.6, respectively) (Sputnik V, AZD1222, or a combination of both) (**Table 3**).

**Table 3:** Humoral immune response to adenovirus-based COVID-19 vaccines according to the vaccination schedule received.

A. Fingolimod			
Schedule	Without antibody response, *n* (%)	With antibody response, *n* (%)	Total
Sputnik V	6 (35.2)	11 (64.8)	17 (100)
AZD1222	11 (47.8)	12 (52.2)	23 (100)
Combined schedule	2 (40)	3 (60)	5 (100)
Total	19	26	45
B. Anti CD20			
Schedule	Without antibody response, *n* (%)	With antibody response, *n* (%)	Total
Sputnik V	6 (85.7)	1 (14.3)	7 (100)
AZD1222	6 (66.6)	3 (33.4)	9 (100)
Combined schedule	2 (66.6)	1 (33.4)	3 (100)
Total	14	5	19

We found no association between the lack of response to antibodies for fingolimod (Chi Square 0.65, p=0.7) (A) or for antiCD20 (Chi Square 0.8, p=0.6) (B) with any particular vaccination scheme (Contingency tables).

**Figure 4:**
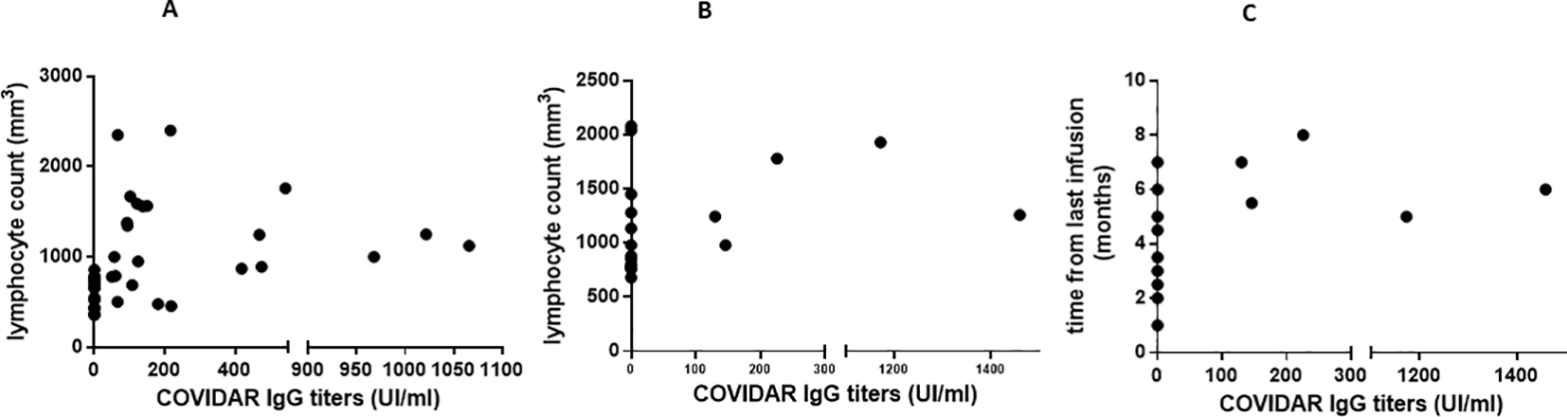
Correlation between absolute lymphocyte count, time of last infusion and antibody titers. We found a correlation between a lower lymphocyte count and a lower antibody titer in patients receiving fingolimod treatment **(A)** (r: 0.67, 95% CI: 0.46- 0.81, *p*=0.0001). In the case of Anti CD20 **(B)** we did not find a statistically significant correlation, but rather a trend (r.0.39, 95% CI -0.08-0.7, *p* = 0.09). The antibody titer was lower when vaccination and last AntiCD20 infusion were closer (r: 0.49, 95% CI: 0.03- 0.7, p = 0.03) **(C)**.

Given that reports from other countries have shown a positive correlation between lower antibody response and lower lymphocyte levels at the time of receiving RNA-based vaccines, we decided to investigate this relationship in our study. Initially, we observed a significantly reduced absolute lymphocyte count in pwMS treated with fingolimod and anti-CD20, compared to other treatments (*p* < 0.0001, one-way ANOVA) (data not shown). Subsequently, a positive correlation was identified between lower lymphocyte count and reduced antibody levels in pwMS receiving fingolimod (*r* = 0.67, 95% CI: 0.46–0.81, *p* ≤ 0.0001) (**Figure 4A**). For those under treatment with anti-CD20, although not statistically significant, there was a trend towards a similar correlation (*r* = 0.39, 95% CI: −0.08–0.7, *p* = 0.09) (**Figure 4B**). Moreover, in these patients, the antibody titer was lower when vaccination and anti-CD20 infusion were administered closely together (*r* = 0.49, 95% CI: 0.03–0.7, *p* = 0.03) (**Figure 4C**).

Regarding MS clinical variables, in the overall population of patients with MS (*n* = 101), no statistically significant differences in the EDSS were found between individuals who showed an antibody response (EDSS: 2.2 ± 1.54) and those who did not (EDSS: 2.94 ± 1.55) (*p* = 0.06). Similarly, there were no disparities in the disease evolution among pwMS who developed an antibody response versus those who did not (*p* = 0.1). Upon examination within the group that elicited an antibody response (*n* = 68), no correlations were discovered between antibody levels and age (rho = 0.1 *p* = 0.9), the interval between vaccine doses (rho = 0.03, *p* = 0.7), years with MS (rho = 0.09 *p* = 0.4), or EDSS scores (rho = 0.08 *p* = 0.4), nor were there significant differences in antibody levels based on the specific vaccination regimens received (*p* = 0.4) (data not shown).

Multivariate regression analysis adjusting for all the clinical variables revealed that the anti-CD20 (β = −0.349, 95% CI: −3,655.6 to −369.01, *p* = 0.017) and fingolimod (β = −0.399, 95% CI: −3,363.8 to −250.9, *p* = 0.023) groups were independent factors associated with low antibody response (*r* ^2^ adjusted = 0.157).

### 3.2 Humoral immune response to the third vaccine dose

In Argentina, pwMS received a third dose at least 1 month after the second dose, known as “additional dose” of primary schedule. Of the total pwMS (*n* = 101) who participated in the study, only 57 patients received the third dose and decided to undergo blood extraction for the determination of anti-Spike antibodies 6 to 8 weeks after this vaccination. **Table 4** shows the characteristics of the recruited population for the third vaccine dose. PwMS received either a homologous schedule (adenovirus-based vaccine as a third dose) or a heterologous schedule (RNA-based vaccine as a third dose). As previously described, 61.5% received a homologous schedule and 38.5% received a heterologous schedule.

**Table 4:** Demographic and clinical characteristics of pwMS who received a third dose of COVID-19 vaccine.

Variable	People with multiple sclerosis (*n* = 57)
Sex (%, n)	
Male Female	64.9% (37) 45.1% (20)
Age (mean, SD)	36.9 (± 11.4)
MS phenotype (%, *n*)	RR 96.5% (55) PP 3.5% (2)
EDSS (mean, SD)	2.9 (± 1.3)
MS evolution in years (mean, SD)	7.3 (± 5.8)
Vaccine schedule (%, *n*)	
Homologous Heterologous	61.5% (35) 38.5% (22)
Days between second and third doses (mean, SD)	81 (± 24.1)
DMT (%, *n*)	Dimethylfumarate 21.05% (12) Fingolimod 42.1% (24) Cladribine 19.29% (11) Anti-CD20* 17.4% (10)

PwMS, people with multiple sclerosis; HC, healthy controls; SD, standard deviation; EDSS, Expanded Disability Status Scale, DMT: disease-modifying therapy, *includes ocrelizumab and rituximab.

Of the total pwMS under fingolimod (*n* = 24), 25% (*n* = 6) still had undetectable anti-Spike antibodies, after receiving a homologous schedule. However, six patients who were non-reactive after the second dose managed to produce antibodies after the third dose, all of them under a homologous schedule. In addition, of the 10 anti-CD20 pwMS recruited, 7 (70%) patients still had no antibody response. However, a pwMS who was non-reactive after the second dose managed to produce antibodies after the third dose, receiving a heterologous regimen. Patients treated with dimethyl fumarate and cladribine showed detectable titers and generated a statistically significant increase after the third dose (*p* = 0.03).

### 3.3 Neutralization analysis

We further evaluated the neutralizing capacity of sera for which a humoral response could be detected, regardless of their treatment status. After the second vaccine dose, anti-Spike IgG titers and neutralizing ID_50_ titers against D614G VOC showed a strong correlation (Spearman *r* = 0.6182; *p* < 0.001). Upon stratifying the correlation analysis by treatment, a significant correlation was observed in the sera of patients treated with dimethyl fumarate (Spearman *r* = 0.8857; *p* < 0.05), whereas no statistically significant correlation was observed for other treatments or controls, likely due to the limited sample size.

**Figure 5:**
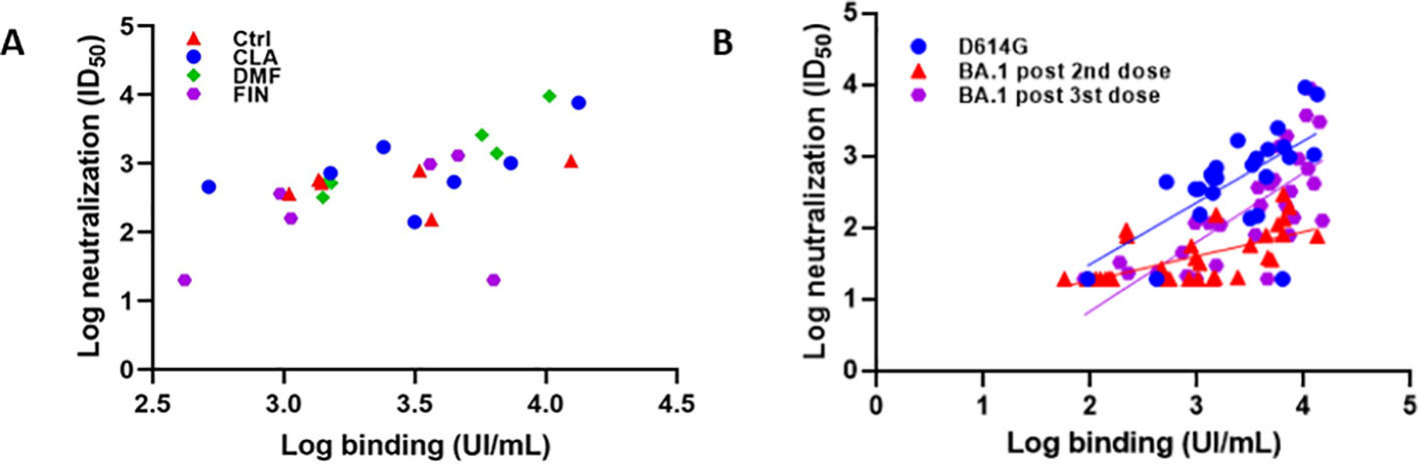
Neutralization tests. **(A)** After the second vaccine dose, anti-Spike IgG titers and neutralizing ID50 titers against D614G VOC showed a strong correlation (Spearman r = 0.6182; p < 0.001). Upon stratifying the correlation analysis by treatment, a correlation was observed in patients treated with DMF (Spearman r = 0.8857; p < 0.05), whereas no statistically significant correlation was observed for other treatments or controls, likely due to the limited sample size. **(B)** After the 2nd dose we found a correlation between total and neutralizing titers against D614G (rho = 0.6; p < 0.001 slope 0.8,95%CI:0.4-1.3). Neutralization capacity was lower for BACI:0.1-0.4). After the 3rd dose, neutralization of BA.1 improved (slope: 0.9 95%CI:0.6-1.2), Ctrl, healthy control; CLA, cladribine; DMF, dimethyl fumarate; FIN, fingolimod.

However, when analyzing the linear regression coefficients among treatments, the slopes of the regression lines are not significantly different from each other, indicating that none of the treatments significantly alter the overall correlation between the two variables [for each correlation, the slope (95% confidence interval) is as follows: IgG second dose vs. D614G ID_50_ = 0.8635 (0.4223 to 1.305); IgG second dose vs. BA.1 ID_50_ = 0.3404 (0.1901 to 0.4908); IgG third dose vs. BA.1 ID_50_ = 0.9682 (0.6622 to 1.274)]. Patients with anti-CD20 were not included in this analysis as there were too few patients with an antibody response to analyze and could not yield significant results (**Figure 5A**).

Additionally, we carried out the PBNA to determine the neutralization capacity of sera after the second and third doses against Omicron BA.1. When we analyzed the correlation between the antibody titers as determined by ELISA and their neutralization capacity against BA.1, we found lower anti-BA.1 ID_50_s, as compared to the Wuhan variant. When we analyzed the neutralization ability to neutralize Omicron in sera from the same group of patients after receiving the third dose, a significant increase in the neutralizing/total Ab ratio was observed (**Figure 5B**).

### 3.4 Cellular immune response

**Figure 6:**
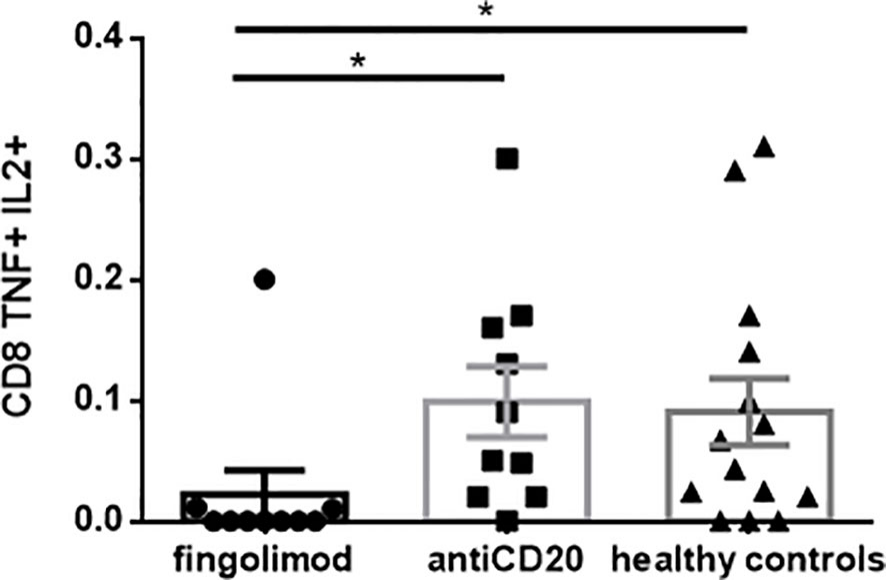
Cellular immune response post 2^nd^ vaccine dose measured by flow cytometry. We found a significantly decreased response in patients treated with fingolimod, compared to patients treated with antiCD20 and healthy controls. We did not find statistically significant differences between healthy controls and patients receiving antiCD20. Kruskal-Wallis test p=0.01, Dunn's post hoc, *p 0.01-0.05, n= 10-14/group.

Regarding T cell-mediated immune responses, we analyzed the proportion of CD8+TNF+IL-2+ T cells in a subgroup of pwMS, since it is one of the most relevant populations presenting a considerable immune response to vaccines. We found a significantly decreased response in patients treated with fingolimod, compared to those treated with anti-CD20 and HC. However, no statistically significant differences were found between HC and patients receiving anti-CD20 (Kruskal–Wallis test *p* = 0.01, Dunn’s *post hoc*, *p = 0.01–0.05, *n* = 10–14/group) (**Figure 6**). Interestingly, CD8+ T-cell responses were stronger and clearer compared to CD4+ T-cell responses, which did not show statistical significance among all groups analyzed (data not shown).

### 3.5 Safety

In this study, we also assessed the safety of the adenovirus-based vaccines. Our results showed that patients with MS had similar rates of adverse reactions to what has been reported in the general population following Sputnik V and AZD1222, suggesting that patients with MS do not show higher risk for vaccine-induced adverse events. Furthermore, the reported adverse events were similar for the three vaccine doses. The most common adverse events were flu-like illness (77.2%, *n* = 78), injection site reactions (76.2%, *n* = 77), headache (75.2%, *n* = 7), and asthenia or fatigue (72.2%, *n* = 73). No patient had an MS relapse. Ten pwMS (9.9%) showed worsening of MS symptomatology that did not even meet criteria for a disease relapse, which was transient in all cases. We found no significant differences in the frequency of adverse events between the two vaccines (Sputnik V versus AZD1222) (data not shown).

## 4 Discussion

Given the limited data on the immunogenicity of adenovirus-based COVID-19 vaccines in LATAM, which were the main vaccines applied in the region, our study aimed to analyze the immune response in this setting. Notably, in the case of Sputnik V, there were no available data on MS.

We found that all HC seroconverted after the second dose of vaccine, while 32.7% of the pwMS did not elicit an antibody response. These results are consistent with findings from RNA-based and inactivated virus vaccines (2, 10, 19, 20, 25). One study compared the antibody response between RNA and adenoviral vaccines (Johnson & Johnson) in pwMS under fingolimod, finding a higher proportion of patients that generated antibodies in those who received the adenoviral vaccine: 55.6% of patients seroconverted with RNA vaccines compared to 83% of patients with the Johnson & Johnson vaccine (26).

When we analyzed the influence of DMT on the antibody response, we found that all pwMS treated with dimethylfumarate and cladribine could generate a humoral immune response. These findings are in line with all the reports performed for these therapies with RNA and inactivated virus vaccines, although there were no reports to this date on adenovirus-based vaccines (2, 10, 16, 25–29). Additionally, no association was found between the absence of seroconversion and the vaccination schedule. Furthermore, we found a lower seroconversion in pwMS under fingolimod and anti-CD20 (ocrelizumab and rituximab) treatments, according to reports from the rest of the world with RNA, inactivated, and adenoviral Johnson & Johnson/ADZ1222 vaccines, although with the latter two mentioned vaccines, the reports are scarce (2, 10, 16, 19, 20, 25, 30–34).

No association was found between seroconversion and the vaccination schedule. However, lower seroconversion rates were observed in patients under fingolimod and anti-CD20 treatments, consistent with global reports on RNA, inactivated virus, and adenoviral vaccines, particularly Johnson & Johnson/ADZ1222.

Given the previously reported low immunogenicity of different vaccines in pwMS, and the high rate of severe COVID-19 in pwMS treated with anti-CD20 mAb (35–37), several studies have analyzed the cellular immune response in these populations, particularly those receiving RNA-based vaccines. Most studies found preserved CD4 and CD8 T-cell immune responses (19, 31, 32, 34, 38–40), despite low seroconversion, and even greater than the T-cell response of HC (40). Further studies are needed to determine whether T-cell responses induced by SARS-CoV-2 vaccination in pwMS receiving anti-CD20 mAb will be sufficient to prevent infection or to reduce the severity of COVID-19 in patients who did not produce anti-SARS-CoV-2 antibodies. Our study is the first to analyze the cellular response against adenovirus-based vaccines in pwMS and demonstrated a preserved CD8+ T-cell response. Based on these results, two hypotheses were proposed. First, in the absence of anti-SARS-CoV-2 antibodies, a significant amount of antigen might drive CD8 T-cell activation and proliferation due to absence of antigen removal by vaccine-induced antibodies. Alternatively, regulatory B cells may directly contribute to reducing CD8 T-cell responses (40).

Regarding fingolimod treatment, no studies have found a preserved cellular immune response to RNA-based vaccines, despite no increased incidence of severe COVID-19 as observed in the anti-CD20 mAb population (16, 19, 30, 35–37).

According to Achiron and colleagues, in this study, we found a correlation between antibody titers and lymphocyte count at the time of vaccination in patients treated with fingolimod, as it was reported for RNA vaccines (2, 10, 30). Additionally, a tendency in the anti-CD20 mAb group was found. However, Tortorella and colleagues did not find this correlation (16). Interestingly, we found that seroconversion was higher when vaccination was furthest from infusion, as reported with the RNA vaccines (10, 34). There are still no reports of this association with adenovector-based vaccines.

Regarding the neutralization capacity of antibodies generated by COVID-19 vaccines in pwMS on different DMTs, there are few reports, all focused on RNA vaccines. Our study revealed a strong correlation between the levels of SARS-CoV-2 specific antibodies and the neutralization capacity against the Wuhan variant, with no significant differences across treatments. Despite the impact of some therapies on antibody-mediated immune response (as evident from ELISA data, particularly with a higher proportion of non-responders in patients treated with fingolimod), the levels of anti-Spike IgG correlate with the neutralizing capacity of sera from those patients, suggesting that the quality of the generated antibodies remains unaltered. A study by Tortorella and colleagues analyzed only patients treated with fingolimod considering the same SARS-CoV-2 variant; they found that only 16.6% of these patients had specific neutralizing antibodies and, in these cases, there was a significant correlation between the neutralizing capacity and antibody titers (16). On the other hand, studies performed in patients with MS on anti-CD20 mAb and fingolimod showed decreased neutralizing capacity of anti-SARS-CoV-2 antibodies compared with HC and pwMS without DMT (17, 41).

The emergence of the Omicron BA.1 variant of SARS-CoV-2 has presented significant challenges to vaccine effectiveness, leading to the need for a third dose.

Studies have consistently demonstrated a reduction in vaccine effectiveness against this variant, emphasizing the importance of a booster dose to enhance protection against Omicron BA.1 (42).

In the case of pwMS, longitudinal research based on mRNA platform vaccines showed that the proportion of patients with MS treated with fingolimod and anti-CD20 mAb therapies achieving seroconversion increases after the third and fourth dose, whereas some patients with MS remain seronegative (43–46). Our results show that, as expected, the neutralization capacity against BA.1 of sera from patients vaccinated with two vaccine doses was significantly reduced as compared to the Wuhan variant, and the neutralizing/total Ab ratio was reduced, which was expected as this particular ELISA uses Wu.1-Spike and RBD as coating antigens. However, we can show that DMT-treated patients who seroconverted reached the same neutralizing/total Ab ratio against BA.1 as HC, confirming that DMT does not influence the quality of antibodies.

Finally, regarding the safety of vaccines, the frequency of adverse events was similar to that reported in the general population for the phase 2 and phase 3 trials of both vaccines analyzed in this work (47, 48).

## 5 Conclusion

Our research provides new data on the immunogenicity of vaccines used in patients with MS that were not previously reported. There is limited information in LATAM on the humoral immune response to COVID-19 vaccines in patients with MS receiving the AZD1222 vaccine. However, the are no data available regarding the Sputnik V vaccine in this population.

Our findings support previous reports on RNA-based vaccines globally, which can assist in the decision-making of neurologists regarding COVID-19 vaccination for patients with MS on DMTs.

## Data Availability

The raw data supporting the conclusions of this article will be made available by the authors, without undue reservation.

## References

[1] Giovannoni G Hawkes C Lechner-Scott J Levy M Waubant E Gold J. The covid-19 pandemic and the use of ms disease-modifying therapies. Multiple Sclerosis Related Disord. (2020) 39:102073. doi: 10.1016/j.msard.2020.102073 PMC7138156 32334820

[2] Achiron A Dolev M Menascu S Zohar DN Dreyer-Alster S Miron S. Covid-19 vaccination in patients with multiple sclerosis: what we have learnt by february 2021. Multiple Sclerosis. (2021) 27:864–70. doi: 10.1177/13524585211003476 PMC8114441 33856242

[3] Schweitzer F Laurent S Fink GR Barnett MH Hartung HP Warnke C. Effects of disease-modifying therapy on peripheral leukocytes in patients with multiple sclerosis. J Neurol. (2021) 268:2379–89. doi: 10.1007/s00415-019-09690-6 PMC8217029 32036423

[4] Longbrake EE Cross AH. Effect of multiple sclerosis disease-modifying therapies on B cells and humoral immunity. JAMA Neurol. (2016) 73:219–25. doi: 10.1001/jamaneurol.2015.3977 26720195

[5] Zrzavy T Kollaritsch H Rommer PS Boxberger N Loebermann M Wimmer I. Vaccination in multiple sclerosis: friend or foe? Front Immunol. (2019) 10:1883. doi: 10.3389/fimmu.2019.01883 31440255 PMC6693409

[6] Vagberg M Kumlin U Svenningsson A. Humoral immune response to influenza vaccine in natalizumab-treated ms patients. Neurol Res. (2012) 34:730–3. doi: 10.1179/1743132812Y.0000000059 22709910

[7] von Hehn C Howard J Liu S Meka V Pultz J Mehta D. Immune response to vaccines is maintained in patients treated with dimethyl fumarate. Neurol(R) Neuroimmunol Neuroinflamm. (2018) 5:e409. doi: 10.1212/NXI.0000000000000409 PMC5688262 29159204

[8] Olberg HK Eide GE Cox RJ Jul-Larsen A Lartey SL Vedeler CA. Antibody response to seasonal influenza vaccination in patients with multiple sclerosis receiving immunomodulatory therapy. Eur J Neurol. (2018) 25:527–34. doi: 10.1111/ene.13537 29205701

[9] Stokmaier D Winthrop K Chognot C Evershed J Manfrini M McNamara J. Effect of ocrelizumab on vaccine responses in patients with multiple sclerosis(2018). Available online at: https://multiple-sclerosis-research.org/2020/03/can-you-respond-to-viral-infections-on-ocrelizumab/ (Accessed November 09, 2023).

[10] Achiron A Mandel M Dreyer-Alster S Harari G Magalashvili D Sonis P. Humoral immune response to covid-19 mrna vaccine in patients with multiple sclerosis treated with high-efficacy disease-modifying therapies. Ther Adv Neurological Disord. (2021) 14:17562864211012835. doi: 10.1177/17562864211012835 PMC8072850 34035836

[11] Tallantyre EC Vickaryous N Anderson V Asardag AN Baker D Bestwick J. Covid-19 vaccine response in people with multiple sclerosis. Ann Neurol. (2022) 91:89–100. doi: 10.1002/ana.26251 34687063 PMC8652739

[12] Achiron A Mandel M Gurevich M Dreyer-Alster S Magalashvili D Sonis P. Immune response to the third covid-19 vaccine dose is related to lymphocyte count in multiple sclerosis patients treated with fingolimod. J Neurol. (2022) 269:2286–92. doi: 10.1007/s00415-022-11030-0 PMC8889521 35235002

[13] Levit E Longbrake EE Stoll SS. Seroconversion after covid-19 vaccination for multiple sclerosis patients on high efficacy disease modifying medications. Multiple Sclerosis Related Disord. (2022) 60:103719. doi: 10.1016/j.msard.2022.103719 PMC8890787 35276450

[14] Dreyer-Alster S Menascu S Mandel M Shirbint E Magalashvili D Dolev M. Covid-19 vaccination in patients with multiple sclerosis: safety and humoral efficacy of the third booster dose. J Neurological Sci. (2022) 434:120155. doi: 10.1016/j.jns.2022.120155 PMC8779784 35091386

[15] Konig M Torgauten HM Tran TT Holmoy T Vaage JT Lund-Johansen F. Immunogenicity and safety of a third sars-cov-2 vaccine dose in patients with multiple sclerosis and weak immune response after covid-19 vaccination. JAMA Neurol. (2022) 79:307–9. doi: 10.1001/jamaneurol.2021.5109 PMC8787678 35072702

[16] Tortorella C Aiello A Gasperini C Agrati C Castilletti C Ruggieri S. Humoral- and T-cell-specific immune responses to sars-cov-2 mrna vaccination in patients with ms using different disease-modifying therapies. Neurology. (2022) 98:e541–e54. doi: 10.1212/WNL.0000000000013108 PMC8826460 34810244

[17] Sabatino JJ Jr. Mittl K Rowles WM McPolin K Rajan JV Laurie MT. Multiple sclerosis therapies differentially affect sars-cov-2 vaccine-induced antibody and T cell immunity and function. JCI Insight. (2022) 7. doi: 10.1172/jci.insight.156978 PMC8876469 35030101

[18] Etemadifar M Sedaghat N Nouri H Lotfi N Chitsaz A Khorvash R. Sars-Cov-2 Serology among People with Multiple Sclerosis on Disease-Modifying Therapies after Bbibp-Corv (Sinopharm) Inactivated Virus Vaccination: Same Story, Different Vaccine. Multiple Sclerosis Related Disord. (2022) 57:103417. doi: 10.1016/j.msard.2021.103417 PMC8607735 34875487

[19] Zabalza A Arrambide G Otero-Romero S Pappolla A Tagliani P Lopez-Maza S. Is humoral and cellular response to sars-cov-2 vaccine modified by dmt in patients with multiple sclerosis and other autoimmune diseases? Multiple Sclerosis. (2022) 28:1138–45. doi: 10.1177/13524585221089540 35475363

[20] Sormani MP Inglese M Schiavetti I Carmisciano L Laroni A Lapucci C. Effect of sars-cov-2 mrna vaccination in ms patients treated with disease modifying therapies. EBioMedicine. (2021) 72:103581. doi: 10.1016/j.ebiom.2021.103581 34563483 PMC8456129

[21] Thompson AJ Banwell BL Barkhof F Carroll WM Coetzee T Comi G. Diagnosis of multiple sclerosis: 2017 revisions of the mcdonald criteria. Lancet Neurol. (2018) 17:162–73. doi: 10.1016/S1474-4422(17)30470-2 29275977

[22] Ojeda DS Gonzalez Lopez Ledesma MM Pallares HM Costa Navarro GS Sanchez L Perazzi B. Emergency response for evaluating sars-cov-2 immune status, seroprevalence and convalescent plasma in Argentina. PloS Pathog. (2021) 17:e1009161. doi: 10.1371/journal.ppat.1009161 33444413 PMC7808630

[23] Lopez MV Vinzon SE Cafferata EGA Nunez FJ Soto A Sanchez-Lamas M. A single dose of a hybrid hadv5-based anti-covid-19 vaccine induces a long-lasting immune response and broad coverage against voc. Vaccines. (2021) 9. doi: 10.3390/vaccines9101106 PMC8537385 34696219

[24] Nie J Li Q Wu J Zhao C Hao H Liu H. Quantification of sars-cov-2 neutralizing antibody by a pseudotyped virus-based assay. Nat Protoc. (2020) 15:3699–715. doi: 10.1038/s41596-020-0394-5 32978602

[25] Ciampi E Uribe-San-Martin R Soler B Garcia L Guzman J Pelayo C. Safety and humoral response rate of inactivated and mrna vaccines against sars-cov-2 in patients with multiple sclerosis. Multiple Sclerosis Related Disord. (2022) 59:103690. doi: 10.1016/j.msard.2022.103690 PMC8842089 35182880

[26] Rommer PS Bsteh G Berger T Zettl UK. Sars-cov-2 antibodies in multiple sclerosis patients depending on the vaccine mode of action? Multiple Sclerosis. (2022) 28:165–7. doi: 10.1177/13524585211039128 34387536

[27] Satyanarayan S Safi N Sorets T Filomena S Zhang Y Klineova S. Differential antibody response to covid-19 vaccines across immunomodulatory therapies for multiple sclerosis. Multiple Sclerosis Related Disord. (2022) 62:103737. doi: 10.1016/j.msard.2022.103737 PMC8916835 35533419

[28] Brill L Rechtman A Zveik O Haham N Levin N Shifrin A. Effect of cladribine on covid-19 serology responses following two doses of the bnt162b2 mrna vaccine in patients with multiple sclerosis. Multiple Sclerosis Related Disord. (2022) 57:103343. doi: 10.1016/j.msard.2021.103343 PMC8539216 35158452

[29] Xia S Duan K Zhang Y Zhao D Zhang H Xie Z. Effect of an inactivated vaccine against sars-cov-2 on safety and immunogenicity outcomes: interim analysis of 2 randomized clinical trials. JAMA. (2020) 324:951–60. doi: 10.1001/jama.2020.15543 PMC7426884 32789505

[30] Iannetta M Landi D Cola G Campogiani L Malagnino V Teti E. B- and T-cell responses after sars-cov-2 vaccination in patients with multiple sclerosis receiving disease modifying therapies: immunological patterns and clinical implications. Front Immunol. (2021) 12:796482. doi: 10.3389/fimmu.2021.796482 35111162 PMC8801814

[31] Katz JD Bouley AJ Jungquist RM Douglas EA O’Shea IL Lathi ES. Humoral and T-cell responses to sars-cov-2 vaccination in multiple sclerosis patients treated with ocrelizumab. Multiple Sclerosis Related Disord. (2022) 57:103382. doi: 10.1016/j.msard.2021.103382 PMC8575541 35158475

[32] Brill L Rechtman A Zveik O Haham N Oiknine-Djian E Wolf DG. Humoral and T-cell response to sars-cov-2 vaccination in patients with multiple sclerosis treated with ocrelizumab. JAMA Neurol. (2021) 78:1510–4. doi: 10.1001/jamaneurol.2021.3599 PMC8461553 34554197

[33] Madelon N Heikkila N Sabater Royo I Fontannaz P Breville G Lauper K. Omicron-Specific Cytotoxic T-Cell Responses after a Third Dose of Mrna Covid-19 Vaccine among Patients with Multiple Sclerosis Treated with Ocrelizumab. JAMA Neurol. (2022) 79:399–404. doi: 10.1001/jamaneurol.2022.0245 35212717 PMC9002341

[34] Rauber S Korsen M Huntemann N Rolfes L Muntefering T Dobelmann V. Immune response to sars-cov-2 vaccination in relation to peripheral immune cell profiles among patients with multiple sclerosis receiving ocrelizumab. J Neurol Neurosurg Psychiatry. (2022) 93(9):978–85. doi: 10.1136/jnnp-2021-328197 35193952 PMC8889453

[35] Alonso R Silva B Garcea O Diaz PEC Dos Passos GR Navarro DAR. Covid-19 in multiple sclerosis and neuromyelitis optica spectrum disorder patients in latin america: covid-19 in ms and nmosd patients in latam. Multiple Sclerosis Related Disord. (2021) 51:102886. doi: 10.1016/j.msard.2021.102886 PMC7937038 33744758

[36] Louapre C Collongues N Stankoff B Giannesini C Papeix C Bensa C. Clinical characteristics and outcomes in patients with coronavirus disease 2019 and multiple sclerosis. JAMA Neurol. (2020) 77:1079–88. doi: 10.1001/jamaneurol.2020.2581 PMC7320356 32589189

[37] Sormani MP Italian Study Group on C-iims. An italian programme for covid-19 infection in multiple sclerosis. Lancet Neurol. (2020) 19:481–2. doi: 10.1016/S1474-4422(20)30147-2 PMC7191287 32359409

[38] Schwarz T Otto C Jones TC Pache F Schindler P Niederschweiberer M. Preserved T cell responses to sars-cov-2 in anti-cd20 treated multiple sclerosis. Multiple Sclerosis. (2022) 28:1041–50. doi: 10.1177/13524585221094478 PMC9131414 35575234

[39] Madelon N Lauper K Breville G Sabater Royo I Goldstein R Andrey DO. Robust T cell responses in anti-cd20 treated patients following covid-19 vaccination: A prospective cohort study. Clin Infect Dis. (2021) 75(1):e1037–e1045. doi: 10.1093/cid/ciab954 PMC8767893 34791081

[40] Apostolidis SA Kakara M Painter MM Goel RR Mathew D Lenzi K. Cellular and humoral immune responses following sars-cov-2 mrna vaccination in patients with multiple sclerosis on anti-cd20 therapy. Nat Med. (2021) 27(11):1990–2001. doi: 10.1038/s41591-021-01507-2 34522051 PMC8604727

[41] Gyang TV Evans JP Miller JS Alcorn K Peng J Bell EH. Neutralizing antibody responses against sars-cov-2 in vaccinated people with multiple sclerosis. Multiple Sclerosis J Experimental Trans Clin. (2022) 8:20552173221087357. doi: 10.1177/20552173221087357 PMC8941285 35342640

[42] Wesemann DR. Omicron’s message on vaccines: boosting begets breadth. Cell. (2022) 185:411–3. doi: 10.1016/j.cell.2022.01.006 PMC8758338 35065712

[43] Milo R Staun-Ram E Karussis D Karni A Hellmann MA Bar-Haim E. Humoral and cellular immune responses to sars-cov-2 mrna vaccination in patients with multiple sclerosis: an Israeli multi-center experience following 3 vaccine doses. Front Immunol. (2022) 13:868915. doi: 10.3389/fimmu.2022.868915 35432335 PMC9012137

[44] Achtnichts L Jakopp B Oberle M Nedeltchev K Fux CA Sellner J. Humoral immune response after the third sars-cov-2 mrna vaccination in cd20 depleted people with multiple sclerosis. Vaccines. (2021) 9. doi: 10.3390/vaccines9121470 PMC8707582 34960216

[45] Wallach AI Schiebel M Picone MA. Antibody response to sars-cov-2 vaccination following typical and three-dose dosing schedules in multiple sclerosis patients treated with disease modifying therapies. Multiple Sclerosis Related Disord. (2022) 63:103856. doi: 10.1016/j.msard.2022.103856 PMC9072817 35636275

[46] Achtnichts L Ovchinnikov A Jakopp B Oberle M Nedeltchev K Fux CA. Sars-cov-2 mrna vaccination in people with multiple sclerosis treated with fingolimod: protective humoral immune responses may develop after the preferred third shot. Vaccines. (2022) 10. doi: 10.3390/vaccines10020341 PMC8875864 35214799

[47] Ramasamy MN Minassian AM Ewer KJ Flaxman AL Folegatti PM Owens DR. Safety and immunogenicity of Chadox1 ncov-19 vaccine administered in a prime-boost regimen in young and old adults (Cov002): A single-blind, randomised, controlled, phase 2/3 trial. Lancet. (2021) 396:1979–93. doi: 10.1016/S0140-6736(20)32466-1 PMC7674972 33220855

[48] Logunov DY Dolzhikova IV Shcheblyakov DV Tukhvatulin AI Zubkova OV Dzharullaeva AS. Safety and efficacy of an rad26 and rad5 vector-based heterologous prime-boost covid-19 vaccine: an interim analysis of a randomised controlled phase 3 trial in Russia. Lancet. (2021) 397:671–81. doi: 10.1016/S0140-6736(21)00234-8 PMC7852454 33545094

